# Whole-exome sequencing reveals *ANO8* as a genetic risk factor for intrahepatic cholestasis of pregnancy

**DOI:** 10.1186/s12884-020-03240-z

**Published:** 2020-09-17

**Authors:** Xianxian Liu, Hua Lai, Xiaoming Zeng, Siming Xin, Liju Nie, Zhenyi Liang, Meiling Wu, Yu Chen, Jiusheng Zheng, Yang Zou

**Affiliations:** 1 Key Laboratory of Women’s Reproductive Health of Jiangxi Province, Jiangxi Provincial Maternal and Child Health Hospital, 330006 Nanchang, Jiangxi China; 2 Central Lab, Jiangxi Provincial Maternal and Child Health Hospital, 330006 Nanchang, Jiangxi China; 3 Department of Obstetrics, Jiangxi Provincial Maternal and Child Health Hospital, 330006 Nanchang, Jiangxi China

**Keywords:** Whole-exome sequencing, *ANO8*, Mutations, Intrahepatic cholestasis of pregnancy

## Abstract

**Background:**

Intrahepatic cholestasis of pregnancy (ICP) is characterized by pruritus and cholestasis in late pregnancy and results in adverse pregnancy outcomes, including preterm delivery and birth weight, which are affected by the genetic and environmental background. However, until now, the genetic architecture of ICP has remained largely unclear.

**Methods:**

Twenty-six clinical data points were recorded for 151 Chinese ICP patients. The data generated from whole-exome sequencing (WES) using the BGISEQ-500 platform were further analyzed by Burrows-Wheeler Aligner (BWA) software, Genome Analysis Toolkit (GATK), ANNOVAR tool, etc. R packages were used to conduct t-test, Fisher’s test and receiver operating characteristic (ROC) curve analyses.

**Results:**

We identified eighteen possible pathogenic loci associated with ICP disease in known genes, covering *ABCB4*, *ABCB11*, *ATP8B1* and *TJP2*. The loci Lys386Gln, Gly527Gln and Trp708Ter in *ABCB4*, Leu589Met, Gln605Pro and Gln1194Ter in *ABCB11*, and Arg189Ser in *TJP2* were novel discoveries. In addition, WES analysis indicated that the gene *ANO8* involved in the transport of bile salts is newly identified as associated with ICP. The functional network of the *ANO8* gene confirmed this finding. *ANO8* contained 8 rare missense mutations that were found in eight patients among the 151 cases and were absent from 1029 controls. Out of the eight SNPs, 3 were known, and the remaining five are newly identified. These variants have a low frequency, ranging from 0.000008 to 0.00001 in the ExAC, gnomAD – Genomes and TOPMED databases. Bioinformatics analysis showed that the sites and their corresponding amino acids were both highly conserved among vertebrates. Moreover, the influences of all the mutations on protein function were predicted to be damaging by the SIFT tool. Combining clinical data, it was found that the mutation group (93.36 µmol/L) had significantly (*P* = 0.038) higher total bile acid (TBA) levels than the wild-type group (40.81 µmol/L).

**Conclusions:**

To the best of our knowledge, this is the first study to employ WES technology to detect genetic loci for ICP. Our results provide new insights into the genetic basis of ICP and will benefit the final identification of the underlying mutations.

## Background

Intrahepatic cholestasis of pregnancy is a pregnancy-related liver disease that mainly occurs in the second and third trimesters of pregnancy and is characterized by pruritus and abnormal liver functions [1]. The symptoms and biochemical abnormalities usually rapidly disappeared after delivery. The incidence of ICP ranges from below 1% to above 15%, with obvious regional and ethnic differences and familial clustering [2]. In China, it also reaches as high as 5.2% [3]. The recurrence rate of ICP in subsequent pregnancies reaches approximately 40% − 60% [1]. ICP increases the risk for adverse pregnancy and perinatal outcomes, including spontaneous preterm birth, intrauterine distress and amniotic fluid fecal infection [4, 5]. The serum bile acid levels in patients increase the risk of adverse perinatal outcomes [6, 7]. Therefore, understanding the molecular basis of ICP disease is very important.

Obviously, ICP is a complex disease that depends on multiple interacting factors, including genetics, endocrine hormones, nutrition and the environment [8]. In recent years, whole-genome and whole-exome sequencing have proven to be powerful new approaches to identify disease-associated variants across the full minor allele frequency (MAF) spectrum in animals [9] and humans [10]. Moreover, the 1000 Genomes Project revealed that rare variants constitute the majority of polymorphic sites in human populations [11]. In particular, accumulating evidence has demonstrated that low-frequency (0.01 ≤ MAF < 0.05) and rare (MAF < 0.01) variations often have a large effect on complex disease etiologies. Increasingly abundant examples of rare variants acting collectively for relevant quantitative traits in medicine have been noted. For example, a previous study revealed that four rare mutations of the *IFIH1* gene act independently on type 1 diabetes (TID) risk [12].

Since the first *ABCB4* mutation in ICP in Caucasians was reported in 1999, the efforts of many researchers have been dedicated to understanding the mechanism of ICP in many different laboratories across Europe [13]. However, deciphering the genetic basis of ICP disease is still a major challenge. To date, only a handful of causative genes (such as *ABCB4* and *ABCB11*) [14] have been identified via genealogical analysis and Sanger sequencing. In recent years, many studies have addressed the role of the *ATP8B1* and *TJP2* genes in ICP susceptibility and identified some possible effect loci associated with ICP [14, 15]. Identification of the association of these genes with ICP disease is helpful to provide timely diagnosis and appropriate medical intervention for ICP pregnant women to avoid adverse maternal and fetal outcomes. Therefore, it is of great importance to identify a large number of ICP susceptibility genes that remain undiscovered.

The anoctamin family contains 10 members (*ANO*1-10) with two major functions: Ca^2+^-dependent ion channels (*ANO1* and *ANO2*) and/or Ca^2+^-activated lipid scramblases with nonselective ion channel activity (*ANO3*-*4*, *ANO6*-*8*) [16–18]. The *ANO* protein family is widely expressed in eukaryotes, exhibits diverse functions in cells throughout the body and is associated with several human diseases [19]. For example, *ANO1* plays roles in membrane excitability in olfactory transduction [19] and affects bile secretion and formation [20]. *ANO8* encodes the transmembrane protein 16H and plays a role in the transport of glucose and other sugars, bile salts and organic acids, metal ions and amine compounds and ion channel transport, according to the functional annotation of the GeneCards. Moreover, Alaish SM et al. previously reported that *ANO8* was differentially expressed in intestinal tissue between AJ (mouse strain) common bile duct ligation (CBDL) and sham-operated mice [21], suggesting that *ANO8* plays a role in hepatobiliary disease. Therefore, we extrapolated and hypothesized that mutations in the *ANO8* gene might affect the protein expression level and thus the transport function of bile salts.

To the best of our knowledge, only a minority of studies have addressed the genetic loci for ICP disease. However, among them, there have been no papers researching ICP with whole-exome sequencing technology. Thus, the objectives of this work were to analyze genetic mutations and putative pathogenic genes associated with clinical data in a sample of 151 Han Chinese individuals with ICP using WES data. A total of 8 mutations in the *ANO8* gene were identified in eight of the 151 individuals.

## Methods

### Samples and clinical features

Peripheral blood samples from 151 Han Chinese ICP patients were collected from the Department of Obstetrics, Jiangxi Provincial Maternal and Child Health Hospital in Nanchang, China. A total of 27 available clinical features, including the age at diagnosis; body mass index (BMI); gestational age; the concentrations of K, Na, Cl, Ca, Mg, and P; white blood cell (WBC), red blood cell (RBC), and platelet (PLT) counts; red blood cell distribution width SD (RDW-SD); alanine transaminase (ALT), aspartate transaminase (AST), total bile acids (TBA), total bilirubin (TBIL), direct bilirubin (DBIL), indirect bilirubin (IDBIL), total cholesterol (CHOL), triglyceride (TG), high-density lipoprotein (HDL), low-density lipoprotein (LDL), and uric acid (UA) levels; newborn birth weight; Apgar score and bleeding amount were recorded. The ion concentration, liver function and lipid index were determined by an AU5800 automatic biochemical analyzer (Beckman Coulter). Routine blood tests were performed using a Sysmex-xn-2000 automatic blood cell analyzer. Summary statistics for all clinical data investigated are shown in Table 1. In addition, 1029 female control individuals without ICP were recruited. Written informed consent was obtained from each participated women in this study.

**Table 1:** Descriptive statistics of 27 clinical data points in 151 Han patients with ICP disease

Features	N	Mean	SD	Min.	Max.
**Basic information**					
Age (years)	151	29.38	5.24	17	43
Gestational age (days)	127	263.43	15.90	215	290
BMI (kg/m^2^)	137	25.79	4.03	19.6	38.5
**Ion Concentration**					
K (mmol/L)	141	4.00	0.31	3.2	4.9
Na (mmol/L)	140	137.44	2.37	132	143
Cl (mmol/L)	140	104.10	2.80	97	112
Ca (mmol/L)	140	2.31	0.15	2	2.9
Mg (mmol/L)	140	0.81	0.15	0.6	1.89
P (mmol/L)	140	1.12	0.18	0.7	1.6
**Routine blood test**					
WBC (× 10^9^)	150	8.56	2.84	4.37	24.23
RBC (× 10^9^)	150	3.84	0.42	2.96	4.98
PLT (× 10^9^)	150	198.39	62.68	75	412
RDW-SD (fL)	150	45.84	4.68	36.2	67.3
**Liver function index**					
ALT (U/L)	139	102.46	127.03	7	595
AST (U/L)	140	86.73	96.28	15	456
TBA (µmol/L)	151	42.99	39.11	4.2	286.8
TBIL (µmol/L)	149	14.88	7.60	5.7	64.8
DBIL (µmol/L)	149	6.45	5.96	0.9	49.5
IDBIL (µmol/L)	149	8.46	3.58	2.9	26.9
**Lipid index**					
CHOL (mmol/L)	144	6.41	1.51	3.35	10.95
TG (mmol/L)	144	3.61	1.56	1.2	11.1
HDL (mmol/L)	144	1.91	0.44	0.92	4.06
LDL (mmol/L)	144	2.86	1.28	0.13	6.28
UA (µmol/L)	141	319.58	81.70	111	574
**Outcomes of pregnant women and newborns**					
Bleeding amount (ml)	114	254.30	103.26	90	810
Apgar score (1–10)	117	9.38	1.08	6	10
Birth weight (kg)	118	3.05	0.75	1.23	5.3

*BMI *Body mass index, *WBC *White blood cell, *RBC *Red blood cell, *PLT *Platelet, *RDW-SD *Red blood cell distribution width SD, *ALT *Alanine transaminase, *AST *Aspartate transaminase, *TBA *Total bile acid, *TBIL *Total bilirubin, *DBIL *Direct bilirubin, *IDBIL *Indirect bilirubin, *CHOL *Total cholesterol, *TG *Triglyceride, *HDL *High-density lipoprotein, *LDL *Low-density lipoprotein, *UA *Uric acid

### Whole-exome sequencing

A total of 151 human genomic DNA samples were isolated from peripheral blood using an Axy Prep Blood Genomic DNA Mini Prep Kit (item No. 05119KC3). DNA quality and concentration were determined by a NanoDrop-1000 spectrophotometer (Thermo Fisher, USA) and gel electrophoresis, respectively. Qualified genomic DNA samples were randomly fragmented, and the size of the library fragments was mainly distributed between 150 bp and 250 bp. End repair of DNA fragments was performed, and an “A” base was added at the 3’-end of each strand. Then, adapters were used to ligate to both ends of the end-repaired/dA-tailed DNA fragments for amplification and sequencing. Amplified DNA fragments were then purified and hybridized to a BGI Exon array. The captured products were then further amplified by circularization. Each qualified captured DNA library was then loaded on BGISEQ-500 platforms. Finally, we obtained the raw sequencing data, which were stored in FASTQ format for each individual. The informatics analysis, mainly including quality control, read mapping, variant calling, filtering and annotation, was conducted by using BWA software, GATK and ANNOVAR tool, respectively.

### Statistical analysis

The t-test method was performed to analyze the potential significant differences between *ANO8* mutations and wild types for the available clinical features. The *P* value is two sided, and the result was considered significantly different at *P* < 0.05. Fisher’s test was conducted to test the significance of differences in frequencies between different groups. In addition, we performed logistic regression for the IPD (individual patient data) analysis to obtain the area under the ROC curve, e.g., AUC, for the association between premature birth and TBA, ALT and AST. All the above-mentioned analyses were carried out with R software.

### Evolutionary conservation analysis

The evolutionary conservation analysis of sites and amino acids was performed in 17 representative vertebrate species, human, chimpanzee, gibbon, macaque, olive baboon, mouse, rat, cow, goat, sheep, pig, dog, dingo, cat, leopard, horse, and elephant, using the genomic alignments of the Ensembl Genome Browser.

## Results

### The WES data results

**Fig. 1:**
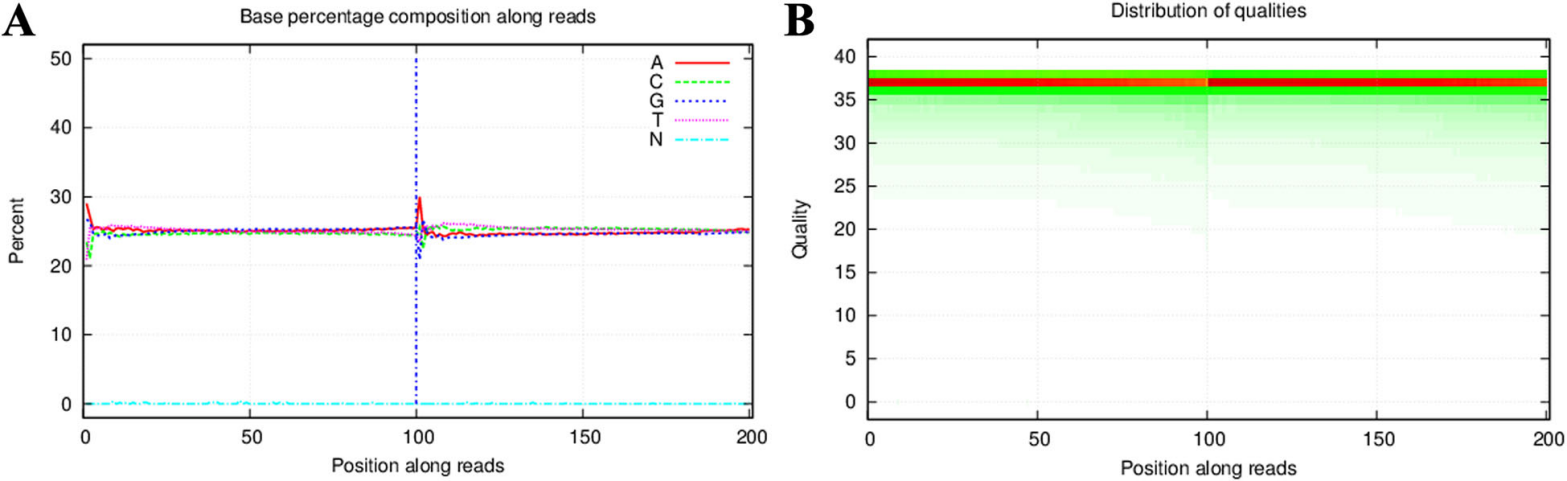
The base percentage composition along reads and distribution of base quality scores on clean reads of ICP66. The X-axis represents positions along reads. The Y-axis is the percent (**a**) and quality value (**b**)

We performed whole-exome sequencing of 151 DNA samples with an average of 14003.98 Mb of raw bases. After removing low-quality reads, we obtained an average of 139,940,436 clean reads (13991.34 Mb). The clean reads of each sample had high Q20 and Q30, which showed high sequencing quality. The average GC content was 51.20%. Figure 1 shows the base percentage composition along reads and the distributions of base quality scores on clean reads of one ICP sample (ICP66). The chromosomal positions of SNPs were based on the UCSC GRCh37/hg19.

We obtained a total of 72,729 variants, including nonsynonymous, missense, splicing, start lost, stop lost/gained variants. First, we excluded variants with MAF ≥ 0.01 from the 1000 Genomes Project (http://www.internationalgenome.org/), ExAC (http://exac.broadinstitute.org/) and dbSNP ((https://www.ncbi.nlm.nih.gov/snp) databases, and 22,956 SNPs were included in subsequent analysis. In addition, 3094 variants were preserved using overlapping methods by the 1029 controls. Then, we ranked the genes and their possible damaging loci using the prediction tool SIFT to assess whether a variant affected protein function. The results implied that the gene *ANO8* was prominent based on its functional annotation related to bile acid transport and pathogenicity prediction of mutations in genes, in addition to the known functional genes *ABCB4*, *ABCB11*, *ATP8B1* and *TJP2*.

### The genetic variants of *ABCB4*, *ABCB11*, *ATP8B1* and *TJP2*

We identified a total of 61 genetic variants, including 46 intron, 6 synonymous, 8 missense, and 1 nonsense variants, in the *ABCB4* gene. Among them, three variants, two missense variants, Lys386Glu and Gly527Glu, and a nonsense variant, Trp708Ter, were novel and reported for the first time. In addition, another two variants, rs1202754797 and rs201502889, were also identified in the *ABCB4* gene (Table 2). For the *ABCB11* gene, we observed five variants: Leu589Met, Gln605Pro, Gln1194Ter, Tyr1130Cys and Arg696Trp. The first three were newly identified mutations. After quality control, we also identified 3 and five possible pathogenic loci in *ATP8B1* and *TJP2*, respectively. The 3 loci were Thr9Met, Gly473Arg and Arg628Trp in *ATP8B1*. The five variants were Arg189Ser, Ala143Thr, Arg324Trp, Arg713Trp and Gly725Glu in the *TJP2* gene. For all the variants, except for Ala268Val, Ala143Thr, Arg324Trp and Gly725Glu, the remaining variants were absent in the 1000 Genomes Project and ExAC databases (Table 2).

**Table 2:** Genetic variants of *ABCB4*, *ABCB11*, *ATP8B1* and *TJP2*

Gene	Patient	Rs#	Chr	Position, bp	Alleles	Protein change	SIFT	MAF in 151 ICP patients	MAF in controls	MAF in 1000G	MAF in ExAC
*ABCB4*	ICP133,135	Novel	7	87,073,053	T/C	Lys386Glu	0.002 (D)	0.0066 [2/(151*2)]	0 [0/(1029*2)]	NP^a^	NP
	ICP154	Novel	7	87,069,134	C/T	Gly527Glu	0.0 (D)	0.0033 [1/(151*2)]	0 [0/(1029*2)]	NP	NP
	ICP21	Novel	7	87,053,310	C/T	Trp708Ter	—	0.0033 [1/(151*2)]	0 [0/(1029*2)]	NP	NP
	ICP97	rs1202754797	7	87,081,001	G/A	Leu216Phe	0.001 (D)	0.0033 [1/(151*2)]	0 [0/(1029*2)]	NP	NP
	ICP153	rs201502889	7	87,079,314	G/A	Ala268Val	0.001 (D)	0.0033 [1/(151*2)]	0 [0/(1029*2)]	0.0002	0.001
*ABCB11*	ICP2	Novel	2	169,826,599	G/T	Leu589Met	0.0 (D)	0.0033 [1/(151*2)]	0 [0/(1029*2)]	NP	NP
	ICP118	Novel	2	169,826,057	T/G	Gln605Pro	0.006 (D)	0.0033 [1/(151*2)]	0 [0/(1029*2)]	NP	NP
	ICP115	Novel	2	169,783,704	G/A	Gln1194Ter	—	0.0033 [1/(151*2)]	0 [0/(1029*2)]	NP	NP
	ICP109	rs1174631566	2	169,787,197	T/C	Tyr1130Cys	0.0 (D)	0.0033 [1/(151*2)]	0 [0/(1029*2)]	NP	NP
	ICP113	rs376216286	2	169,820,808	G/A	Arg696Trp	0.004 (D)	0.0033 [1/(151*2)]	0 [0/(1029*2)]	NP	NP
*ATP8B1*	ICP134	rs150268416	18	55,399,014	G/A	Thr9Met	0.003 (D)	0.0033 [1/(151*2)]	0 [0/(1029*2)]	NP	NP
	ICP85	rs781746896	18	55,355,543	C/T	Gly473Arg	0.0 (D)	0.0033 [1/(151*2)]	0 [0/(1029*2)]	NP	NP
	ICP43	rs752045131	18	55,338,750	G/A	Arg628Trp	0.001 (D)	0.0033 [1/(151*2)]	0 [0/(1029*2)]	NP	NP
*TJP2*	ICP75	Novel	9	71,835,934	G/T	Arg189Ser	0.005 (D)	0.0033 [1/(151*2)]	0 [0/(1029*2)]	NP	NP
	ICP26	rs144396411	9	71,833,267	G/A	Ala143Thr	0.004 (D)	0.0033 [1/(151*2)]	0 [0/(1029*2)]	0.00059	0.003
	ICP7	rs189916909	9	71,836,337	C/T	Arg324Trp	0.014 (D)	0.0033 [1/(151*2)]	0 [0/(1029*2)]	0.000399	0.001
	ICP132	rs760622082	9	71,851,917	C/T	Arg713Trp	0 (D)	0.0033 [1/(151*2)]	0 [0/(1029*2)]	NP	NP
	ICP96	rs201366118	9	71,851,954	G/A	Gly725Glu	0.007 (D)	0.0033 [1/(151*2)]	0 [0/(1029*2)]	0.0002	0.001

^a^*NP *Not present in the database

### The eight variants of the *ANO8* gene

In addition, interestingly, we found that a total of eight missense mutations in the *ANO8* gene in eight out of the 151 samples from patients with ICP disease (Table 3). Three of these eight mutations are known SNPs, namely, rs1316267732, rs760834212 and rs1391524054. They were identified in 30-, 23- and 24-year-old patient samples. The 30-year-old patient (ICP66) with a high TBA level (129.30 µmol/L) underwent one spontaneous abortion and had two children. The remaining five variants are novel, namely rs1, rs2, rs3, rs4 and rs5. The patients who carried rs1, rs2 and rs3 gave birth to their babies by cesarean section, while the other two patients had spontaneous abortions. Excluding the two spontaneous abortions, three out of 5 pregnant women gave birth prematurely (gestational age < 37 weeks).

**Table 3:** Descriptive statistical analysis of basic information of eight patients

ICP	SNP	Additional variants^a^	Age (years)	Gestational age (weeks)	BMI (kg/m^2^)	TBA (µmol/L)	Gravidity (times)	Parity (times)	Type of delivery
ICP66	rs1316267732	No	30	38 + 1	24.8	129.3	3	1	Cesarean
ICP64^b^	rs1	No	26	33 + 6	33.3	47.6	1	0	Cesarean
ICP40	rs2	No	33	35 + 1	22.1	185.5	3	1	Cesarean
ICP158	rs3	No	30	38 + 3	21.0	37.5	3	0	Cesarean
ICP50	rs760834212	No	23	35 + 6	26.4	120.8	2	0	Cesarean
ICP28	rs1391524054	No	24	40 + 1	22.8	14.8	1	0	Vaginal delivery
ICP151	rs4	No	31	31 + 3	27.4	78.2	2	1	Spontaneous abortion
ICP148	rs5	No	31	17	25.1	133.2	3	1	Spontaneous abortion

^a^ The patient did not contain any possible pathogenic mutations in the *ABCB4*, *ABCB11*, *ATP8B1* and *TJP2* genes

^b^ The new SNPs are marked with a gray background

In addition, 122 women of the 151 sampled women delivered their babies. Out of the 122 women, ninety individuals (73.3%, 90/122) gave birth by cesarean section, whereas 32 (26.7%, 32/122) gave birth by vaginal delivery. Thirty-two (26.7%, 32/122) delivered their babies prematurely, and 17 infants’ (13.9%, 17/122) birth weights were below 2.5 kg. Three of the 6 babies were born preterm.

### Sanger sequencing to validate *ANO8* variants and an additional 1029 control individuals

**Fig. 2:**
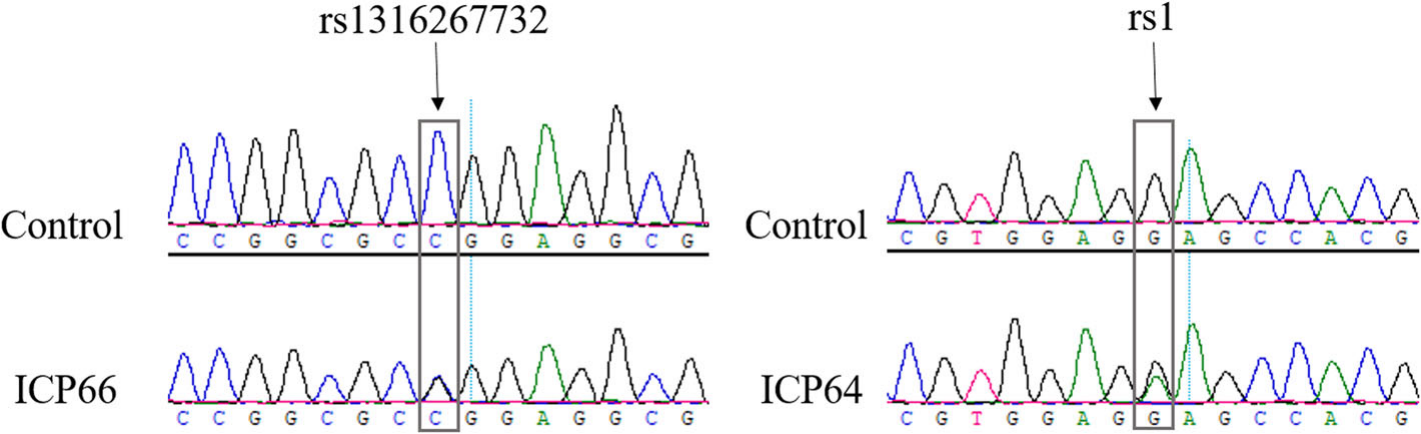
The sequencing electropherograms of rs1316267732 and rs1 mutations in the *ANO8* gene. The mutation location is marked with an arrow

A total of six pairs of primers (Table 4) were designed to amplify PCR products. Then, a comparative analysis of missense mutations of the *ANO8* gene was conducted by DNA sequencing from eight ICP patients and an additional 1029 control individuals with WES sequencing. Figure 2 shows the sequencing electropherograms of the known SNP rs1391524054 and the novel mutation rs1.

**Table 4:** Six pairs of primers used to sequence the 8 missense variants of the human ANO8 gene

Rs#	PCR product (bp)	Forward primer (5’-3’)	Reverse primer (5’-3’)
rs1316267732	309	GCCTTTGTCTCCTCCTCCCG	CCAGGTTACGTTTGACCCTGAT
rs1	501	GACTGAGACCCACTTGTCCC	ACACCTCTCTGCCTTTGCTC
rs2	529	TTCTACTACCCGCCCTGGAA	CTGTCCGATGGTGGTGACTC
rs3	390	ATCACCCGCCAGTTCCTCCA	TTCCTCGCCCTCCTCCTCGT
rs760834212	578	CATGATTCTGGTGGCCGAGA	AGCTTGTGACCTGAGCCTTC
rs1391524054	578	CATGATTCTGGTGGCCGAGA	AGCTTGTGACCTGAGCCTTC
rs4	530	GCCTTTATGTGCCTGGATGC	CGCCCCTGTGAATGACTGAT
rs5	530	GCCTTTATGTGCCTGGATGC	CGCCCCTGTGAATGACTGAT

### Assessing the functional impact of rare*ANO8* variants

These eight *ANO8* mutations were absent from the 1000 Genomes Project and 1029 local controls from our hospital. Additionally, the MAFs of these mutations were low, ranging from 8e-6 to 1e-5 in three databases, e.g., ExAC, gnomAD – Genomes and TOPMED. Using Fisher’s test method, we found no significant differences in the frequencies of the variants between the 151 cases and 1029 controls (*P* = 0.13); in contrast, the frequencies in the databases were significantly different. This relatively lower significance between cases and controls (*P* = 0.13) than between cases and databases might be due to the number of samples involved.

Furthermore, we evaluated the influence of these eight mutations on protein function by using the web-available tool SIFT (http://sift.bii.a-star.edu.sg/) and obtained a score. As a SIFT score less than 0.05 is considered damaging, an amino acid substitution with such a score would be detrimental to the function of *ANO8*. We found that all these variants were predicted to be damaging (Table 5).

**Table 5:** Eight rare *ANO8* missense variants in the databases

Rs#	Chr	Position, bp	Alleles	Protein change	SIFT	MAF in 151 ICP cases	MAF in 1029 controls	MAF in ExAC	MAF in gnomAD - Genomes	MAF in TOPMED	*P* value (cases-controls)	*P* value (cases-ExAC)	*P* value (cases- gnomAD - Genomes)	*P* value (cases-TOPMED)
rs1316267732	19	17,445,461	G/C	Gly7Arg	0.002 (D)	0.0033	0	NP	0.00001 NP		0.13	—	0.0043	—
rs1	19	17,441,657	G/A	Glu325Lys	0.041 (D)	0.0033	0	NP	NP	NP	0.13	—	—	—
rs2	19	17,440,988	G/C	Val407Leu	0.022 (D)	0.0033	0	NP	NP	NP	0.13	—	—	—
rs3	19	17,439,584	G/T	Ser538Ile	0.039 (D)	0.0033	0	NP	NP 0.000030		0.13	—	—	0.0097
rs760834212	19	17,435,864	C/T	Ala998Val	0.004 (D)	0.0033	0	0.000058	0.000064	0.000008	0.13	0.01	0.01	0.0024
rs1391524054	19	17,435,780	G/C	Ser1026Thr	0.0 (D)	0.0033	0	NP	NP	0.000008	0.13	—	—	0.0024
rs4	19	17,434,670	G/T	Ala1119Ser	0.008 (D)	0.0033	0	NP	0.00004	0.000072	0.13	—	0.01	0.012
rs5	19	17,434,537	C/T	Ala1163Val	0.005 (D)	0.0033	0	NP	NP	NP	0.13	—	—	—

See the footnotes in Tables 2 and 3

### Evolutionary conservation analysis

**Fig. 3:**
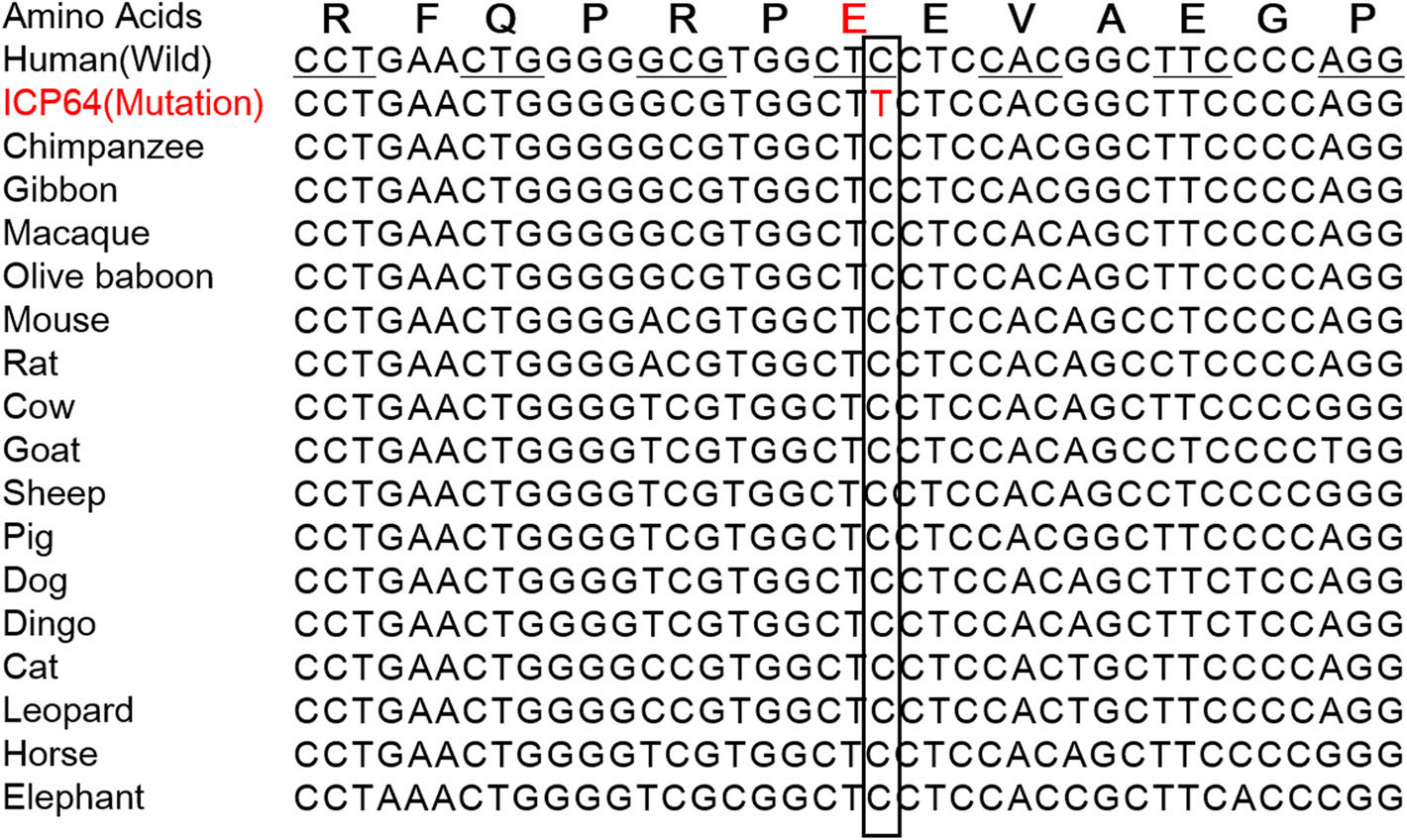
Evolutionary conservation analysis of the *ANO8 *rs1 (p. Glu325Lys) mutation. The single nucleotide C (indicating the complementary G allele) in the black solid line and the triple nucleotide CTC (indicating the GAG codon encoding proline) in the black horizontal line are highly conserved among vertebrates

Evolutionary conservation analysis showed that the rs1 site wild-type nucleotide allele (C) and its corresponding amino acid (proline) were both highly conserved among vertebrates, e.g., pigs, cows, sheep, dogs and cats (Fig. 3).

### Tissue expression

We used the human base website (https://hb.flatironinstitute.org) to predict *ANO8* gene expression, function, regulation, and interactions in humans. The gene expression results showed that *ANO8* was expressed in liver tissue with reasonable confidence (0.71). This result is consistent with the findings of the NCBI (https://www.ncbi.nlm.nih.gov/gene/57719) and GeneCards (https://www.genecards.org/cgi-bin/carddisp.pl?gene=ANO8&keywords=ANO8) websites regarding the expression of *ANO8* in the liver.

**Fig. 4:**
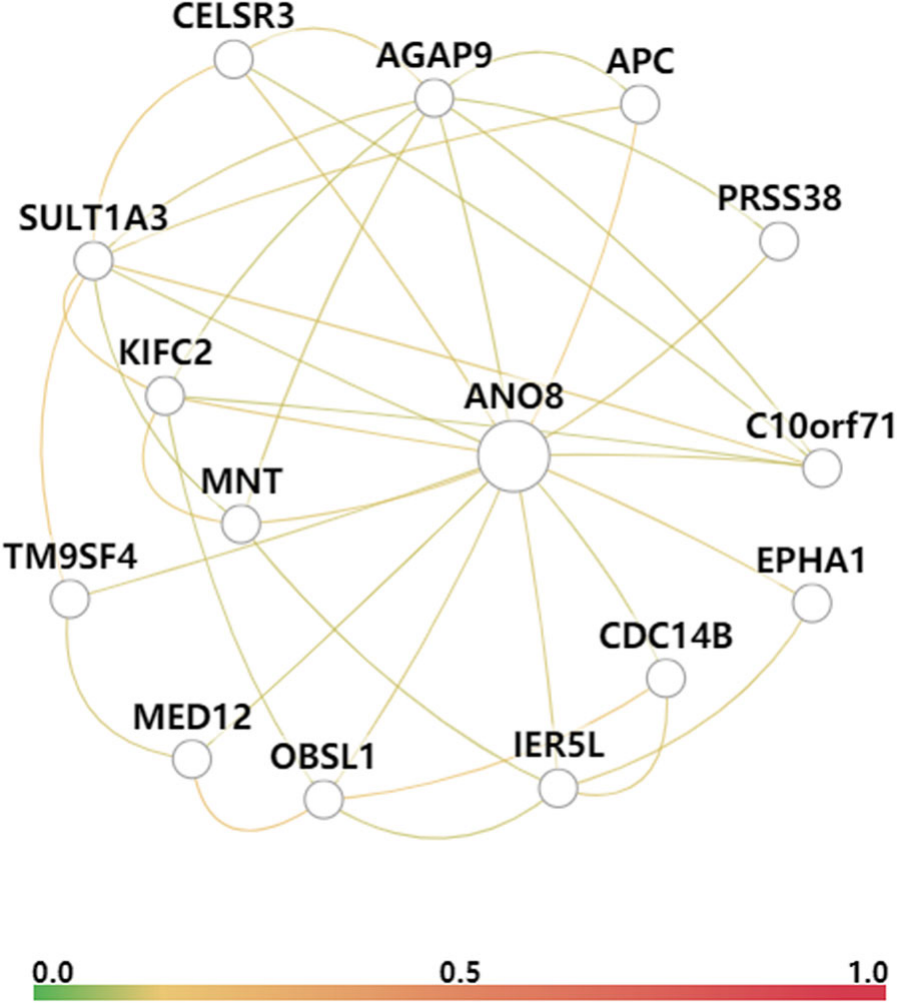
The functional networks of the *ANO8* gene in liver tissue. The color between genes represents the average weight of connections to the query genes

To simultaneously analyze the function of *ANO8*, we further explored the biological process of *ANO8*, including the transport of inorganic anions, anions and chloride and the transmembrane transport of the above three ions. In addition, a functional network that captured liver tissue-specific interactions covering 5 data types, namely, coexpression, interaction, TF binding, GSEA microRNA targets and GSEA perturbations, from large data compendia was produced (Fig. 4). The results showed that the genes in the functional network were relevant to transport, such as *EPHA1* [22], *CELSR3*, *C10orf71*, *CDC14B*, *TM9SF4* [23], and the Wnt signaling pathway, including *APC* [24], *IER5L*, *OBSL1*, and *MED12* [25], suggesting that the function of the *ANO8* protein was likely to be related to the transport of bile salts.

### Correlations between mutations and clinical data

In the 151 ICP samples, regardless of whether the difference was significant, the mutation group tended to be associated with higher Ca^2+^ concentrations, platelet counts, TBA levels, TG levels, and bleeding amounts and lower birth weights (Table 6). Notably, the mutation group had significantly (*P* = 0.038) a higher TBA level than the wild-type group. Moreover, that of the mutation group (93.36 µmol/L) was 2-fold greater than that of the wild-type group (40.81 µmol/L).

**Table 6:** The potential correlation of *ANO8* mutations with clinical and laboratory data in samples from 151 Han Chinese patients with ICP disease

Features^a^	ICP without *ANO8* mutations	ICP with *ANO8* mutations	*P* value
Basic information			
Age (years, mean ± SD, N^b^)	29.43 ± 5.31 (*n* = 143)	28.5 ± 3.43 (*n* = 08)	0.62
BMI (kg/m^2^)	25.82 ± 3.37 (*n* = 129)	25.36 ± 3.62 (*n* = 08)	0.71
Laboratory, mean (range, N)			
K (mmol/L)	4.00 (3.20–4.90, 134)	3.94 (3.6–4.3, 7)	0.61
Na (mmol/L)	137.47 (132.00–143.00, 133)	136.71 (134.00–139.00, 7)	0.41
CL (mmol/L)	104.17 (97.00–112.00, 133)	102.71 (100.00–104.00, 7)	0.17
Ca (mmol/L)	2.31 (2.00–2.90, 133)	2.33 (2.23–2.48, 7)	0.71
Mg (mmol/L)	0.81 (0.60–1.89, 133)	0.79 (0.70–0.87, 7)	0.36
P (mmol/L)	1.12 (0.72–1.60, 133)	1.04 (0.70–1.30, 7)	0.21
WBC (× 10^9^)	8.61 (4.37–24.23, 142)	7.75 (5.90–10.03, 8)	0.40
RBC (× 10^12^)	3.85 (2.96–4.98, 142)	3.59 (3.25–4.02, 8)	0.08
PLT (× 10^9^)	197.61 (75.00–412.00, 142)	212.25 (112.00–328.00, 8)	0.52
RDW-SD (fL)	45.93 (36.20–67.30, 142)	44.30 (39.70–49.30, 8)	0.34
TBA (µmol/L)	40.81 (4.20–286.80, 143)	93.36 (14.80–185.50, 8)	0.038
CHOL (mmol/L)	6.41 (3.35–10.95, 137)	6.40 (4.91–8.75, 7)	0.98
TG (mmol/L)	3.59 (1.20–10.44, 137)	3.88 (1.56–11.10, 7)	0.82
Birth weight (kg)	3.06 (1.23–5.30, 112)	2.86 (2.45–3.35, 5)	0.36
Bleeding amount (ml)	251.74 (90.00 − 810.00, 109)	310.00 (190.00–600.00, 5)	0.48

^a^See the footnotes in Table 1

^b^N: total number

**Fig. 5:**
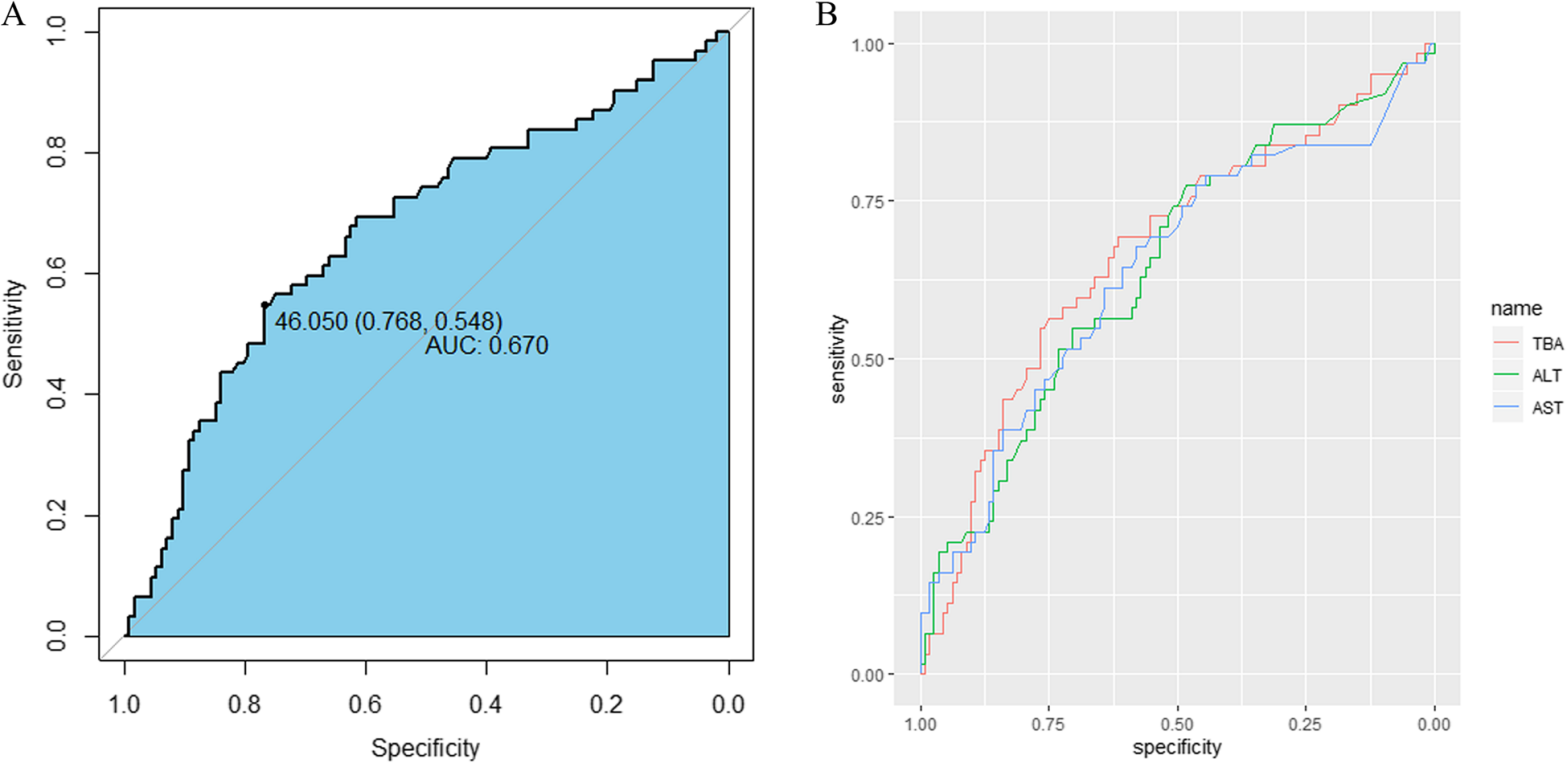
ROC curves for the association between premature birth and serum biochemical markers. **a** Association between premature birth and TBA level. **b** Association between premature birth and TBA, ALT, and AST levels

Moreover, TBA measured by fasting peripheral blood of pregnant women is an important indicator of ICP diagnosis. The IPD analysis (Fig. 5) showed that the TBA level was more highly predictive of premature birth (AUC: 0.670 [95% CI 0548-0.768]) than the ALT and AST levels. The preterm delivery need increased at a TBA cut-off value of 46.05 µmol/L.

## Discussion

So far, most reseachers make efforts to dissect the genetic architecture of ICP disease primarily focusing on *ABCB4*. Previously, three studies simultaneously identified *ABCB4* Ile237Ile (rs2109505) as significantly associated with ICP [26–28]. These loci were also detected in our population. The MAFs in the 1000 Genomes Project and ExAC databases were 0.26 and 0.27, respectively. We hypothesize that this locus contributes to disease susceptibility by linkage disequilibrium between rs2109505 and the causative variant. Our study confirmed the role of *ABCB11* and further expanded the role of *ABCB11* gene which encoded the bile salt export pump. Our result confirmed previous studies have shown that the presence of Arg696Trp mutation in ICP population. In addition to the Arg696Trp mutation, other three novel mutations, including one prematurely stop codon Gln1194Ter, and two missense mutations Gln605Pro and Leu589Pro, were predicted as pathogenic. Besides, we did not identify any loci corresponding to *ATP8B1* and *TJP2* in the previous ICP literature [14, 29, 30], a reasonable explanation for this discrepancy may be the distinct genetic background and genetic heterogeneity of the populations.

Combined with clinical data, we found that 80.79% (122/151) delived the baby, in which, 26.7% (32/122) birthed prematurely and 13.9% (17/122) of the newborns weighted less than 2.5 kg. Similarily, the eight patients with *ANO8* mutations having 3 newborns delivered prematurely and two spontaneous abortion The above results suggested that women with ICP had increased adverse perinatal outcome incidences, e.g., premature birth, abortion and reduced birth weight, which was consistent with the results of previous studies [2, 31]. Besides, the eight patients with *ANO8* variants did not carry the possible potential effect loci of the known functional genes, *ABCB4*, *ABCB11*, *ATP8B1* and *TJP2*, for ICP disease, implying that these ICP cases with *ANO8* mutations are not caused by these mutation of functional known genes.

Bioinformatics analysis suggested that these eight variants in *ANO8* gene might play an important role in the etiology of ICP disease. However, ICP disease is regulated by multiple rare variants independently or aggregatively, and further experimental verification is needed. For example, a previous study [32] employed exome array analysis to identify five new loci and low-frequency variants influencing insulin processing and secretion. Cohen et al. [33] reported that the aggregation of multiple rare variants has been associated with reduced sterol absorption and plasma low-density lipoprotein levels.

Based on the expression and function results of *ANO8* combined with literature reports, the function of *ANO8* was likely to be related to the transport of bile salts in the liver. Therefore, the mutations in the *ANO8* gene identified in ICP women could cause bile acid transport disorder, which leads to bile acid accumulation in liver tissue. Of course, it is noteworthy that the role of the *ANO8* gene and its mutations in cholestasis of pregnancy is based on bioinformatics analysis derived from WES data and network data. It remains to be determined whether *ANO8* mutations cause structural and functional defects in *ANO8*. Therefore, subsequent cell function and in vivo experiments for *ANO8* are particularly important.

Compared with wild-type group, we found that mutation group of *ANO8* gene has higher TBA levels, TG levels and lower birth weights, suggesting that these mutations of the *ANO8* gene might be positively involved in the pathogenesis of ICP disease. In addition, recent studies have also reported that TBA levels ≥ 40 µmol/L increased the risk of perinatal complications, such as low Apgar scores, stillbirth and preterm labor [6, 34, 35], which was consistent with our result, e.g. TBA level of 46.05 µmol/L were a critical value in increasing preterm labor.

## Conclusions

In conclusion, by whole-genome sequencing analysis, we identified 18 possible pathogenic loci associated with ICP in the *ABCB4*, *ABCB11*, *ATP8B1* and *TJP2* genes, seven of which were novel loci. Furthermore, 8 missense mutations, including 3 known and five novel mutations, were detected in the *ANO8* gene in eight of 151 Han ICP patients. To the best of our knowledge, this study is the first report revealing mutations for ICP disease by WES. By Sanger sequencing, conservation analysis, and protein functional prediction analysis, we confirmed that these variants existed and were associated with ICP. Further research should target the molecular mechanisms of these mutations in ICP disease. Our study provides new insights into the genetic architecture of ICP disease and may contribute to ICP genetic diagnosis.

## Data Availability

The datasets used and/or analyzed during the current study are available from the corresponding author on reasonable request.

## Abbreviations

ICP: Intrahepatic cholestasis of pregnancy
WES: Whole-exome sequencing
BWA: Burrows-Wheeler Aligner
GATK: Genome Analysis Toolkit
ROC: Receiver operating characteristic
MAF: Minor allele frequency
WBC: White blood cell
RBC: Red blood cell
PLT: Platelet
RDW-SD: Red blood cell distribution width SD
ALT: Alanine transaminase
AST: Aspartate transaminase
TBA: Total bile acid
TBIL: Total bilirubin
DBIL: Direct bilirubin
IDBIL: Indirect bilirubin
CHOL: Total cholesterol
TG: Triglyceride
HDL: High-density lipoprotein
LDL: Low-density lipoprotein
UA: Uric acid
IPD: Individual patient data
